# Neighborhood Conditions, Self-Management Abilities, and Psychological Well-Being Among Chinese Older Adults

**DOI:** 10.1093/geroni/igab046.1136

**Published:** 2021-12-17

**Authors:** Keqing Zhang, Bei Wu, Wei Zhang

**Affiliations:** 1 University of Hawaii at Manoa, Honolulu, Hawaii, United States; 2 New York University, New York, New York, United States

## Abstract

Abstract Few studies have examined the associations between neighborhood conditions and psychological well-being for Chinese older adults in the U.S. This study examined how neighborhood conditions were associated with psychological well-being through self-management abilities as a pathway among Chinese older adults in Hawaiʻi. Survey data were collected in 2018 and ordinary Least Square regressions and mediation analysis were conducted. For the whole sample, both neighborhood physical conditions and social cohesion were significantly associated with psychological well-being, and the associations were significantly mediated by self-management abilities. The foreign-born subsample shared similar results with the whole sample. For the U.S.-born subsample, psychological well-being was only significantly associated with neighborhood physical conditions, and the association was mediated by self-management abilities. Our findings suggest that both physical and social neighborhood conditions are associated with psychological well-being, particularly for foreign-born older adults, and psychological resources such as self-management abilities could mediate the associations.

